# Monitoring for Constipation Following Clozapine Titration in a Working-Age Adult Inpatient Setting: A Retrospective Audit

**DOI:** 10.1192/bjo.2026.11802

**Published:** 2026-06-30

**Authors:** Ameer Noor, Faryal Rana

**Affiliations:** 1 South West London & St George’s NHS Mental Health Trust, London, United Kingdom; 2 Surrey and Borders Partnership NHS Foundation Trust, Guildford, United Kingdom

## Abstract

**Aims:**

Clozapine-induced gastrointestinal hypomotility (CIGH) is a serious and potentially life-threatening adverse effect, with constipation representing an early warning sign. During clozapine titration, clinical attention may be disproportionately focused on haematological monitoring, risking under-recognition of gastrointestinal side effects. This audit aimed to assess adherence to Trust guidelines for monitoring constipation following clozapine titration in a working-age adult inpatient setting. It was hypothesised that formal completion of Glasgow Antipsychotic Side Effect Scale for Clozapine (GASS-C) at 30 days post-titration would be limited. Secondary objectives were to explore whether constipation was identified and documented through alternative clinical means and whether appropriate treatment was initiated when constipation was present.

**Methods:**

A retrospective audit was undertaken across working-age adult inpatient wards at Farnham Road Hospital. Patients registered with the Clozaril Patient Monitoring Service between October 2023 and October 2024 were identified with support from the hospital pharmacy clozapine service. Patients aged over 65 years and those maintained on an established dose of clozapine were excluded. Electronic patient records were reviewed to determine whether GASS-C had been completed at 30 days following clozapine titration, whether constipation was documented elsewhere in clinical notes at that time point, and whether laxatives had been prescribed.

**Results:**

Fourteen patients were identified, of whom eight met inclusion criteria (six male and two female). GASS-C was completed at the 30-day endpoint in three patients (37.5%). Constipation was documented in the clinical notes of six patients (75%), and seven patients (87.5%) were prescribed laxatives. The modal clozapine dose reached at 30 days was 300 mg.

**Conclusion:**

This audit demonstrates that formal monitoring for constipation using GASS-C following clozapine titration is inconsistently implemented. However, constipation was commonly identified and treated through routine clinical documentation, suggesting awareness of risk despite poor adherence to the recommended monitoring tool. Embedding GASS-C into clozapine titration workflows may support more consistent and systematic monitoring. A re-audit is planned to assess the impact of these interventions.

